# Plasma anion gap and risk of in-hospital mortality in patients with spontaneous subarachnoid hemorrhage

**DOI:** 10.3389/fneur.2022.1008030

**Published:** 2022-10-06

**Authors:** LinJin Ji, Xin Tong, KaiChun Wang, ZhiQun Jiang, Aihua Liu

**Affiliations:** ^1^Department of Neurosurgery, The First Affiliated Hospital of Nanchang University, Nanchang, China; ^2^Beijing Neurosurgical Institute and Beijing Tiantan Hospital, Capital Medical University, Beijing, China

**Keywords:** subarachnoid hemorrhage, anion gap, intensive care unit, in-hospital mortality, ICU mortality

## Abstract

**Background:**

The association between the serum anion gap (AG) and prognosis of patients with spontaneous subarachnoid hemorrhage (SAH) remains unknown. Thus, this study aimed to explore the association between AG levels and mortality in patients with SAH in the intensive care unit (ICU).

**Methods:**

This was a retrospective analysis of data stored in the Medical Information Mart for Intensive Care–IV and eICU Collaborative Research databases. Critically ill patients diagnosed with spontaneous SAH were included. The primary outcome measure was in-hospital all-cause mortality. A multivariate Cox proportional hazards regression model and a restricted cubic spline were used to evaluate the relationship between AG concentration and outcomes. Kaplan–Meier curves were used to compare cumulative survival among patients with AG levels.

**Results:**

A total of 1,114 patients were enrolled. AG concentration was significantly associated with in-hospital all-cause mortality [hazard ratio ([HR], 1.076 (95% confidence interval (CI), 1.021–1.292; *p* = 0.006)]. The risk of mortality was higher in the Category 2 group (AG ≥10 mmol/L and <13 mmol/L; HR, 1.961; 95% CI, 1.157–3.324; *p* = 0.0) and the Category 3 group (AG ≥13 mmol/L; HR, 2.151; 95% CI, 1.198–3.864; *p* = 0.010) than in the Category 1 group (AG < 10 mmol/L). Cumulative survival rates were significantly lower in patients with higher AG levels (log-rank *p* < 0.001).

**Conclusions:**

In-hospital and ICU mortalities increase with increasing AG concentration in patients with SAH. An increased serum AG level is an independent, significant, and robust predictor of all-cause mortality. Thus, serum AG levels may be used in the risk stratification of SAH.

## Introduction

Spontaneous subarachnoid hemorrhage (SAH) accounts for 5–10% of all strokes (1). Patients with SAH tend to be younger than patients with other stroke subtypes, thus leading to an enormous burden of premature mortality (2). Half of surviving SAH patients experience long-term neuropsychological complications and lower quality of life (3). Early identification and appropriate treatment regimens can improve the overall survival of patients with SAH. Thus, a robust and easily accessible clinical indicator for determining prognosis is needed for patients with SAH.

The plasma anion gap (AG) is a mathematical derivation parameter calculated using the formula Na^+^+ (Cl^−^ +HCO^3−^). AG has been widely applied in diagnosing various forms of metabolic acidosis for more than 50 years (4). Previous research has found relationships between AG and mortality in patients with many different diseases, such as acute renal failure (5), cerebral infarction (6), acute myocardial infarction (7), acute ischemic stroke (8), coronary artery disease (9), and aortic aneurysms (10). Furthermore, in the general population, which is essentially free of these diseases, higher levels of AG might be of prognostic significance because an increase in AG has been associated with insulin resistance (11), hypertension (12), and low cardiorespiratory fitness (13). However, it is still unknown whether such changes in AG during the course of SAH are associated with a risk difference in mortality. Therefore, this study aimed to investigate the relationship between AG and SAH using publicly accessible clinical databases.

## Methods

### Study design and population

This retrospective study analyzed data from the Medical Information Mart for Intensive Care (MIMIC)-IV database (version:1.0) (14) and eICU Collaborative Research Database (15). The MIMIC-IV database, as an update to the MIMIC-III database, contains de-identified health-related data of over 40,000 unique patients who were admitted to the critical care units of Beth Israel Deaconess Medical Center between 2008 and 2019. The eICU database is a multicenter database (208 hospitals) comprising de-identified health data associated with over 200,000 ICU admissions between 2014 and 2015 across the United States. Adult patients with spontaneous SAH, as defined according to the International Classification of Diseases, Ninth and Tenth Revision (ICD-9 and ICD-10) codes and Acute Physiology and Chronic Health Evaluation admission codes, were included in the study. Patients meeting the following criteria were excluded: (1) age <18 or >90 years, (2) with insufficient AG data, and (3) with diagnoses related to traumatic SAH. One of the authors who passed the Collaborative Institutional Training Initiative exam and accessed database for data extraction (X.T. certification number:43334826).

### Data extraction and outcome measures

The following patient characteristics were collected: (1) comorbidities including myocardial infarct, congestive heart failure, peripheral vascular disease, chronic pulmonary disease, rheumatic disease, peptic ulcer disease, liver disease, diabetes, renal disease, and malignant cancer; (2) Sequential Organ Failure Assessment (SOFA) score; (3) first day vital signs, including temperature, systolic blood pressure, diastolic blood pressure, and respiratory rate heart rate; (4) and first day laboratory test results, including AG, bicarbonate, creatinine, chloride, glucose, hematocrit, hemoglobin, lactate, platelets, potassium, sodium, blood urea nitrogen (BUN), white blood cell (WBC), and calcium. The overall Charlson Comorbidity Index (CCI) (16) was calculated using 18 categories of medical conditions identified in the medical records. For patients with multiple intensive care unit (ICU) admissions, we collected information only on the first ICU admission.

The primary outcome measure was in-hospital mortality, and the secondary outcome measure was ICU mortality. The patients were classified into three groups based on the three AG categories (Category 1, AG <10 mmol/L; Category 2, AG ≥ 10 mmol/L and <13 mmol/L; and Category 3, AG ≥ 13 mmol/L). Previous studies have shown that an increase in AG levels is associated with poor clinical outcomes in several diseases. To thoroughly analyze whether this trend also occurred in patients with SAH, we only collected the minimum results for patients with a multi-lab test in the first 24 h of ICU admission.

### Statistical analysis

Continuous variables were presented as the mean and standard deviation, while categorical variables were presented as proportions in each category, substratified by AG concentrations. Chi-square or Fisher's exact tests were used to compare categorical variables, and the *t*-test or one-way analysis of variance was used to compare continuous variables. Multivariate Cox proportional hazards regression models adjusted for potential confounders were used to assess hazard ratios (HRs) of mortality for AG concentration in SAH patients. Three cox models were performed as follows: model 1, without adjusting for any confounders; model 2, adjusting for demographic information, vital signs and score system which have *p* values < 0.2 in the univariate analysis; model 3, adjusting for all confounders with *p* values < 0.2 in the univariate analysis. Restricted cubic spline (RCS) models fitted with three knots at the 10^th^, 50^th^, and 90^th^ percentiles of AG were used for multivariate Cox proportional hazards regression model 3 to show the association between AG and in-hospital mortality in patients with SAH. The Kaplan–Meier method was employed to calculate the absolute risk of in-hospital and ICU mortalities for each subgroup of different AG concentrations. The data were reported as HRs with 95% confidence intervals (CIs). All statistical analyses were performed using R software (version 4.0.1). All tests were two sided, and statistical significance between two or more groups was set at *p* < 0.05.

## Results

### Patient characteristics

**Figure 1:**
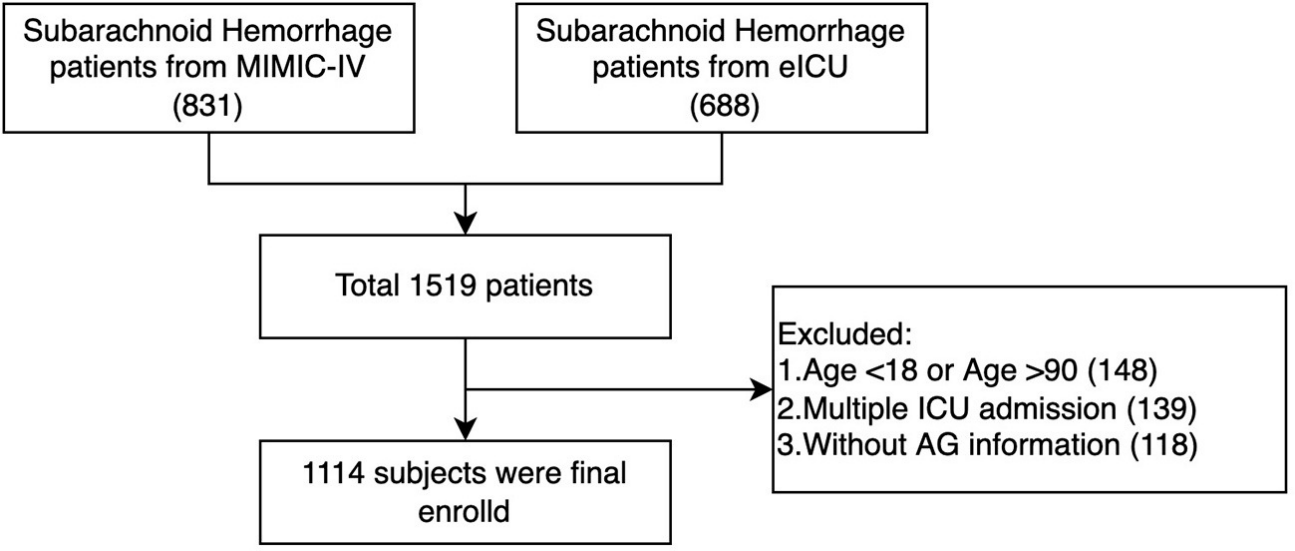
Flow charts of patient inclusion.

A total of 1,519 patients were confirmed to have spontaneous SAH, including 831 patients from the MIMIC-IV database and 688 patients from the eICU database. After exclusion, 1,114 patients were finally included in the analysis (Figure 1). The mean age of the total cohort was 58.74 ± 14.51 years, and 42.5% (474/1,114) were men. The in-hospital mortality and ICU mortality rates were 17.4% (194/1,114) and 13.2% (147/1,114), respectively. The basic patient characteristics by category are summarized in Table 1. The mean AG concentrations of the total cohort and Categories 1, 2, and 3 were 11.84 ± 3.70 mmol/L, 7.06 ± 1.75 mmol/L, 11.73 ± 1.09 mmol/L, and 16.72 ± 2.18 mmol/L, respectively. In general, patients with higher AG levels also had higher in-hospital (14.1 vs. 16.8 vs. 25.6%, *p* = 0.006) and ICU mortality (10.2 vs. 12.2 vs. 19.9%, *p* = 0.003). Patients with higher AG levels also had larger vital signs indices and a higher proportion of chronic pulmonary disease, diabetes, paraplegia and renal disease.

**Table 1:** Patient characteristics by anion gap concentration subgroup.

**Characteristics**	**Anion gap subgroups (mmol/L)**			***P* value**
	**Category 1**	**Category 2**	**Category 3**	
	**(<10, *n* = 283)**	**(≥10, <14; *n* = 476)**	**(≥14; *n* = 236)**	
Age, years [mean (SD)]	57.32 (15.60)	59.52 (14.13)	59.10 (14.42)	0.125
Male (%)	123 (43.5)	192 (40.3)	108 (45.8)	0.359
Days in Hospital [mean (SD)]	14.34 (19.74)	13.16 (10.52)	11.95 (10.94)	0.149
Days in ICU [mean (SD)]	8.32 (7.37)	9.17 (8.89)	7.90 (7.73)	0.115
Hospital mortality (%)	40 (14.1)	80 (16.8)	58 (24.6)	0.006
ICU mortality (%)	29 (10.2)	58 (12.2)	47 (19.9)	0.003
Vital signs [mean (SD)]				
Heart rate	61.69 (12.78)	63.27 (12.49)	64.03 (15.34)	0.121
Systolic blood pressure	97.41 (15.46)	97.68 (14.72)	99.97 (19.54)	0.144
Diastolic blood pressure	49.45 (9.86)	48.11 (10.13)	49.80 (11.54)	0.075
Respiratory rate	11.39 (4.02)	11.90 (3.58)	12.49 (3.75)	0.005
Temperature	36.25 (0.78)	36.40 (0.60)	36.39 (0.71)	0.013
SPO2	91.82 (10.70)	93.05 (7.44)	90.58 (11.91)	0.007
Comorbidities [*n* (%)]				
Myocardial infarction	4 (1.4)	20 (4.2)	11 (4.7)	0.072
Congestive heart failure	7 (2.5)	22 (4.6)	15 (6.4)	0.097
Peripheral vascular disease	6 (2.1)	17 (3.6)	15 (6.4)	0.04
Chronic pulmonary disease	15 (5.3)	57 (12.0)	25 (10.6)	0.01
Rheumatic disease	2 (0.7)	6 (1.3)	5 (2.1)	0.367
Peptic ulcer disease	1 (0.4)	3 (0.6)	2 (0.8)	0.765
Mild liver disease	6 (2.1)	12 (2.5)	4 (1.7)	0.774
Severe liver disease	1 (0.4)	4 (0.8)	1 (0.4)	0.648
Diabetes	7 (2.5)	36 (7.6)	19 (8.1)	0.008
Renal disease	15 (5.3)	13 (2.7)	19 (8.1)	0.006
Paraplegia	4 (1.4)	30 (6.3)	15 (6.4)	0.005
Malignant cancer	8 (2.8)	9 (1.9)	5 (2.1)	0.694
Charlson Comorbidity Index [mean (SD)]	3.62 (1.81)	4.04 (2.01)	4.20 (2.13)	0.002
SOFA score [mean (SD)]	3.16 (2.80)	3.49 (2.69)	3.68 (2.74)	0.083
Laboratory results [mean (SD)]				
Anion gap	7.06 (1.75)	11.73 (1.09)	16.72 (2.18)	<0.001
Bicarbonate	23.23 (3.32)	22.68 (3.31)	20.57 (3.18)	<0.001
Creatinine	0.83 (0.48)	0.78 (0.39)	1.03 (1.67)	0.002
Chloride	104.29 (4.81)	103.49 (4.72)	102.12 (4.70)	<0.001
Glucose	120.78 (33.32)	127.32 (35.57)	139.60 (50.88)	<0.001
Hematocrit	35.80 (5.85)	35.81 (4.95)	36.79 (5.60)	0.052
Hemoglobin	11.99 (2.11)	12.02 (1.80)	12.35 (1.98)	0.062
Platelets	210.84 (70.99)	211.63 (71.56)	232.58 (89.81)	0.001
Potassium	3.66 (0.49)	3.63 (0.43)	3.72 (0.51)	0.042
Sodium	137.41 (3.96)	138.34 (4.23)	138.12 (3.63)	0.008
BUN	13.88 (9.22)	13.24 (7.12)	17.50 (18.63)	<0.001
WBC count	10.95 (13.11)	10.72 (3.92)	12.08 (4.58)	0.09
Calcium	8.23 (0.86)	8.31 (0.73)	8.51 (0.74)	<0.001

SAH, subarachnoid hemorrhage; SD, standard deviation; BUN, blood urea nitrogen; WBC, white blood cell.

### Association between AG and outcomes

**Figure 2:**
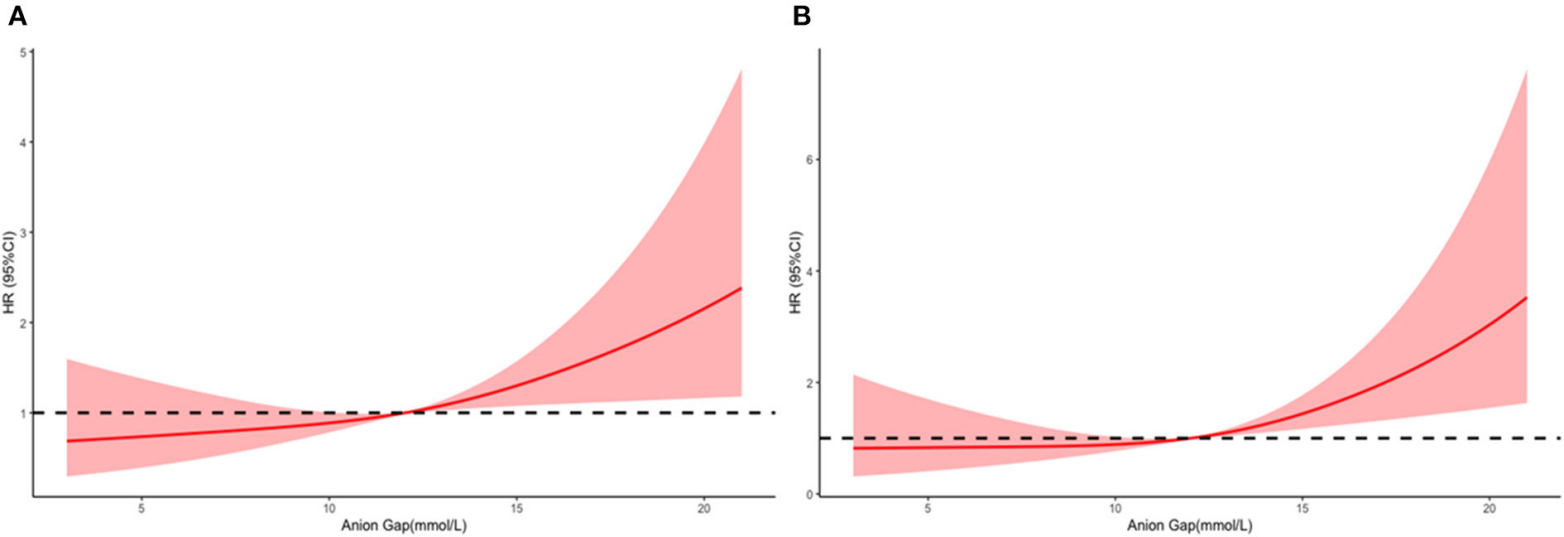
Multivariable adjusted hazard ratios (HRs) for all-cause mortality according to levels of anion gap (AG) on a continuous scale (mmol/L). Solid deep red lines are multivariable-adjusted HRs. Light red areas are the 95% confidence intervals derived from restricted cubic spline regressions with 3 knots. Dashed black lines are reference lines for no association at a hazard ratio of 1.0. In general, in-hospital **(A)** and ICU **(B)**, mortality are increased as AG concentration increased. In-hospital mortality analysis is adjusted for age, systolic blood pressure, diastolic blood pressure, respiratory rate, temperature, SPO2, mild liver disease, serve liver disease, renal disease, malignant cancer, Charlson Comorbidity Index, SOFA score, bicarbonate, creatinine, glucose, hematocrit, hemoglobin, platelets, potassium, BUN, WBC count, and calcium. ICU mortality analysis is adjusted for age, heart rate, systolic blood pressure, diastolic blood pressure, respiratory rate, temperature, SPO2, mild liver disease, serve liver disease, myocardial infarction, paraplegia, renal disease, malignant cancer, Charlson Comorbidity Index, SOFA score, bicarbonate, creatinine, glucose, hemoglobin, platelets, potassium, BUN, WBC count, and calcium.

**Figure 3:**
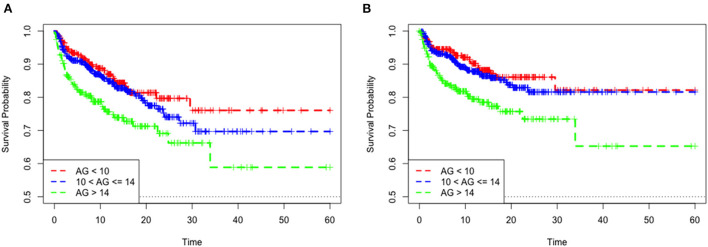
Kaplan–Meier survival curve of mortality among quartile groups of serum anion gap. In-hospital mortality **(A)**. ICU mortality **(B)**.

The mean hospital stay duration for the survivor and the non-survivor groups was 14.76 ± 14.40 days and 6.75 ± 7.08 days, respectively. Hazards ratios (HR) of these three models were as follows: model 1, 1.061 (95%CI, 1.022–1.102; *p* = 0.002); model 2, 1.060 (95%CI, 1.015–1.105; *p* = 0.007) (adjusting for age, sofa score, systolic blood pressure, diastolic blood pressure, respiratory rate, temperature and SPO2); model 3, 1.076 (95%CI, 1.021–1.292; *p* = 0.006) (adjusting for age, systolic blood pressure, diastolic blood pressure, respiratory rate, temperature, SPO2, mild liver disease, serve liver disease, renal disease, malignant cancer, Charlson Comorbidity Index, SOFA score, bicarbonate, creatinine, glucose, hematocrit, hemoglobin, platelets, potassium, BUN, WBC count, and calcium) (Table 2). RCS curve showed that the risk of in-hospital mortality increased as the AG concentration increased (Figure 2A). With the Category 1 group as the reference, the HRs for all-cause in-hospital mortality were higher in the Category 2 (HR, 1.961; 95% CI, 1.157–3.324; *p* = 0.012) and Category 3 groups (HR, 2.151; 95% CI, 1.198–3.386; *p* = 0.010) (Table 3). Similar results were found for the association between AG concentration and ICU mortality (Figure 2B and Table 3). The Kaplan-Meier survival curve demonstrated significantly lower cumulative survival for patients with higher AG levels (log-rank *p* < 0.001) (Figure 3).

**Table 2:** Association between the anion gap and in-hospital and ICU mortalities.

**Characteristics**	**HR (95% CI)**	***P* value**
**In-hospital mortality**		
Model 1^a^	1.061 (1.022–1.102)	0.002
Model 2^b^	1.060 (1.015–1.105)	0.007
Model 3^c^	1.076 (1.021–1.292)	0.006
**ICU mortality** ^**b**^		
Model 1^a^	1.078 (1.032–1.125)	<0.001
Model 2^b^	1.022 (1.007–1.037)	0.011
Model 3^d^	1.095 (1.030–1.164)	0.003

^a^Adjusting for nothing.

^b^Adjusting for age, heart rate, systolic blood pressure, diastolic blood pressure, respiratory rate, temperature, SPO2 and SOFA score.

^c^Adjusting for age, systolic blood pressure, diastolic blood pressure, respiratory rate, temperature, SPO2, mild liver disease, serve liver disease, renal disease, malignant cancer, Charlson Comorbidity Index, SOFA score, bicarbonate, creatinine, glucose, hematocrit, hemoglobin, platelets, potassium, BUN, WBC count, and calcium.

^d^Adjusting for age, heart rate, systolic blood pressure, diastolic blood pressure, respiratory rate, temperature, SPO2, mild liver disease, serve liver disease, myocardial infarction, paraplegia, renal disease, malignant cancer, Charlson Comorbidity Index, SOFA score, bicarbonate, creatinine, glucose, hemoglobin, platelets, potassium, BUN, WBC count, and calcium.

**Table 3:** Multivariate Cox analysis for in-hospital and ICU mortalities across anion gap groups.

**Characteristics**	**HR (95% CI)**	***P* value**
**In-hospital mortality** ^**a**^		
Category 1	Ref	Ref
Category 2	1.961 (1.157–3.324)	0.012
Category 3	2.151 (1.198–3.864)	0.010
**ICU mortality** ^**b**^		
Category 1	Ref	Ref
Category 2	1.869 (1.007–3.467)	0.047
Category 3	2.553 (1.279–5.10)	0.008

^a^Adjusting for age, systolic blood pressure, diastolic blood pressure, respiratory rate, temperature, SPO2, mild liver disease, serve liver disease, renal disease, malignant cancer, Charlson Comorbidity Index, SOFA score, bicarbonate, creatinine, glucose, hematocrit, hemoglobin, platelets, potassium, BUN, WBC count, and calcium.

^b^Adjusting for age, heart rate, systolic blood pressure, diastolic blood pressure, respiratory rate, temperature, SPO2, mild liver disease, serve liver disease, myocardial infarction, paraplegia, renal disease, malignant cancer, Charlson Comorbidity Index, SOFA score, bicarbonate, creatinine, glucose, hemoglobin, platelets, potassium, BUN, WBC count, and calcium.

## Discussion

The relationship between serum AG and clinical outcomes in patients with spontaneous SAH is still unknown. This study found that patients with increased serum AG levels had worse clinical outcomes and a greater probability of in-hospital and ICU deaths.

Serum AG has been widely used to identify errors in measuring serum electrolytes or to detect and evaluate various acidotic conditions. Most recently, high AG levels were reported to be associated with decreased clinical outcomes in several diseases (5–13). Thus, serum AG, as a low-cost and easily available clinical index, may have great potential for evaluating prognosis. SAH accounts for 5–10% of strokes, but it occurs at a younger age, resulting in more loss of productive life years. The fatality rate ranges from 25 to 50%, owing to the consequences of either original bleeding or serious complications (16). In this study, the total in-hospital mortality rate was relatively low (17.7%). This explains why we only collected data on the first ICU admission, and this estimate did not fully account for patients who died before receiving medical attention (17).

Previous studies have also reported that case fatality rates have decreased with the introduction of improved management strategies (16). We found that the risk of mortality increased with increasing AG levels. This result may be partly explained by a decrease in bicarbonate levels. Increased plasma AG often reflected an acid imbalance due to inadequate tissue perfusion and renal excretion function disorders in the current study. Acid-base balance is critical for optimal physiological functions and cell metabolism (18). Categories 1, 2, and 3 had mean bicarbonate concentrations of 23.23 ± 3.32, 22.68 ± 3.31, and 20.57 ± 3.18 mmol/L (*p* < 0.001), respectively, and 5.3, 2.7, and 8.1% (*p* = 0.008) of patients had renal disorders, respectively. Cerebral vasospasm to extravascular blood cells and delayed cerebral ischemia are two of the most important and common complications in SAH (19).

Cerebral vasospasm and delayed cerebral ischemia may lead to cerebral ischemia or brain hypoxia. These could be direct or indirectly cause by excess lactate production in the tissue, causing hyperventilation and hypocapnia (20) that could increase vasoconstriction and decrease intracranial pressure (21). Although the mechanism by which hypocapnia affects the prognosis of brain injury remains unclear, several studies have reported that hypocapnia is associated with poor outcomes in patients with brain injury. Solaiman et al. found that the duration of hypocapnia was associated with symptomatic vasospasm and unfavorable outcomes in aneurysmal SAH patients (22). Cai et al. also reported that both hypercapnia and hypocapnia were associated with a higher mortality risk in patients with SAH or other craniocerebral diseases (23). However, high AG levels may reflect renal excretion function disorders. In a large consecutive series of prospective cohort studies, Zacharia et al. found that renal dysfunction was an independent predictor of worse outcomes in patients with aneurysmal SAH patients (24).

Another explanation might be that the increase in AG levels partly reflects the increase in sodium concentration in SAH patients (25). Previous studies have reported that hypernatremia is associated with poor outcomes in SAH patients (26–32). In a clinical trial conducted at 54 neurosurgical centers in North America, Qureshi et al. found that although hypernatremia was not associated with the risk of symptomatic vasospasm, it was independently associated with poor outcomes after adjusting for previously identified outcome predictors (30). Fisher et al. found that hypernatremia was associated with adverse cardiovascular and neurological outcomes (26). Kumar et al. also found that hypernatremia was a significant risk factor for acute kidney injury in a patient with SAH (32). These results support that in addition to hypernatremia, AG levels may also further increase.

Compared to established indicators such as blood gas analysis or lactate, plasma AG is less costly and more frequently available in low-resource settings (33). As an alternative to assess acid-base imbalances, blood gas analysis may be used to predict prognosis in critically ill patients. However, blood gas analysis can indeed be influenced by compensatory respiratory alkalosis. Plasma AG is a sensitive tool for the treatment of metabolic diseases. Plasma AG is straightforward and does not require arterial puncture. In this study, plasma AG was found to be an independent predictor of in-hospital and ICU mortalities in patients with SAH.

## Limitations

Our study has a few limitations that should be mentioned. First, the retrospective design may have introduced patient selection and analysis bias. However, we used real-world data from two large databases with patients from more than 200 hospitals to improve the generalizability of the results as much as possible. Second, patients with SAH were diagnosed using administrative diagnostic codes. Although we only selected the primary diagnosis sequence, there was still a chance of misclassification, leading to faulty connections. Third, hypoalbuminemia was prevalent in critically ill patients, which might have led to AG underestimation. Consequently, we only collected the minimum result for AG in the first 24 h to evaluate the relationship between AG increase and mortality. Third, the MIMIC-IV and eICU databases did not include long-term follow-up events. Thus, the association between long-term functional results and AG levels in patients was unclear. Given the small window of time for therapy following symptom onset of cerebral hemorrhage and the increase in cerebral hemorrhage severity, some patients with SAH may not have been referred to other institutions. Therefore, our study may not have included high-risk patients. Furthermore, individuals with mild cerebral hemorrhage may have been admitted to the general ward and excluded from our study. Further research is needed to evaluate the external generalizability of our findings. Additional prospective case-control data are also needed to demonstrate the relevance of AG as a clinical marker for predicting the outcomes of SAH.

## Conclusions

This large population-based study shows that the risk of mortality increases as AG concentration increases in patients with SAH. AG is an independent risk factor for all-cause in-hospital and ICU mortalities and is associated with poor clinical outcomes in these patients. Therefore, plasma AG could be a valuable marker for evaluating the prognosis of critically ill patients with SAH.

## Data availability statement

The original contributions presented in the study are included in the article/supplementary material, further inquiries can be directed to the corresponding authors.

## Ethics statement

Ethical review and approval was not required for the study on human participants in accordance with the local legislation and institutional requirements. Written informed consent from the patients/participants or patients/participants' legal guardian/next of kin was not required to participate in this study in accordance with the national legislation and the institutional requirements.

## Author contributions

ZJ and AL conceived and designed the study. XT and KW collected the data. XT and LJ conceived of the project, analyzed the data, and wrote the paper. All authors read and approved the final manuscript.

## Funding

This study was supported by the Nanchang Science and Technology Support Program (No: 2020-133-24). This research was supported by the cultivation foundation of the First Affiliated Hospital of Nanchang University (No. YFYPY202038).

## Conflict of interest

The authors declare that the research was conducted in the absence of any commercial or financial relationships that could be construed as a potential conflict of interest.

## Publisher's note

All claims expressed in this article are solely those of the authors and do not necessarily represent those of their affiliated organizations, or those of the publisher, the editors and the reviewers. Any product that may be evaluated in this article, or claim that may be made by its manufacturer, is not guaranteed or endorsed by the publisher.
